# Supplementary material for: GR-AttNet: Robotic grasping with lightweight spatial attention mechanism
Source: PLoS One. 2025 Dec 4;20(12):e0337599. doi: 10.1371/journal.pone.0337599 (PMC12677535; doi:10.1371/journal.pone.0337599)
Supplement: Attachment — Submitted filename: PONE-D-25-28907-reviewed.pdf [file pone.0337599.s001.pdf]

PLOS ONE

GR-AttNet: Robotic Grasping with Lightweight Spatial Attention Mechanism

--Manuscript Draft--

**Manuscript Number:**	PONE-D-25-28907
**Article Type:**	Research Article
**Full Title:**	GR-AttNet: Robotic Grasping with Lightweight Spatial Attention Mechanism
**Short Title:**	GR-AttNet: Robotic Grasping with Lightweight Spatial Attention Mechanism
**Corresponding Author:**	Shengbang Zhou<br>Nanning Normal University<br>nanning, guangxi CHINA
**Keywords:**	
**Abstract:**	Robotic grasping is crucial in manufacturing, logistics, and service robotics, but existing methods struggle with object occlusion and complex arrangements in cluttered scenes. We propose the Generative Residual Attention Network (GR-AttNet), based on the Generative Residual Convolutional Neural Network (GR-CNN). The spatial attention mechanism enhances its adaptability to clutter, and architectural optimization maintains high accuracy while reducing parameters and boosting efficiency. Experiments show GR-AttNet achieves 98.1% accuracy on the Cornell Grasping Dataset, 94.9% on the Jacquard Dataset, with processing speeds of 148 ms and 163 ms respectively, outperforming several state-of-the-art methods. With only 2.8 million parameters, it surpasses various existing models. Simulations further validate over 50% success rates across different scenario complexities. However, challenges remain in handling small object missed detection and severe occlusion cases. GR-AttNet offers a novel, highly practical solution for robotic grasping tasks.
**Order of Authors:**	Shengbang Zhou
	zhongsheng liu
	Chuanqi Li
	Shutian Liu
	Yadong Liu
**Additional Information:**	
**Question**	**Response**
**Financial Disclosure**<br><br>Enter a financial disclosure statement that describes the sources of funding for the work included in this submission. Review the submission guidelines for detailed requirements. View published research articles from *PLOS ONE* for specific examples.<br><br>This statement is required for submission and **will appear in the published article** if the submission is accepted. Please make sure it is accurate.	Yes

Powered by Editorial Manager® and ProduXion Manager® from Aries Systems Corporation

**Funded studies**<br>Enter a statement with the following details:<br>• Initials of the authors who received each award<br>• Grant numbers awarded to each author<br>• The full name of each funder<br>• URL of each funder website<br>• Did the sponsors or funders play any role in the study design, data collection and analysis, decision to publish, or preparation of the manuscript?<br><br>Did you receive funding for this work?	
Please add funding details.<br>as follow-up to "**Financial Disclosure**<br><br>Enter a financial disclosure statement that describes the sources of funding for the work included in this submission. Review the submission guidelines for detailed requirements. View published research articles from *PLOS ONE* for specific examples.<br><br>This statement is required for submission and **will appear in the published article** if the submission is accepted. Please make sure it is accurate.<br><br>**Funded studies**<br>Enter a statement with the following details:<br>• Initials of the authors who received each award<br>• Grant numbers awarded to each author<br>• The full name of each funder<br>• URL of each funder website<br>• Did the sponsors or funders play any role in the study design, data collection and analysis, decision to publish, or preparation of the manuscript?<br><br>Did you receive funding for this work?"	Guangxi Key Technologies R&D Program (No.AB241484046) and GuangxiScience and Technology Program (Project No. GUIKEAB23075177)
Please select the country of your main research funder (please select carefully as in some cases this is used in fee calculation).	CHINA - CN

Powered by Editorial Manager® and ProduXion Manager® from Aries Systems Corporation

as follow-up to "**Financial Disclosure**<br><br>Enter a financial disclosure statement that describes the sources of funding for the work included in this submission. Review the submission guidelines for detailed requirements. View published research articles from *PLOS ONE* for specific examples.<br><br>This statement is required for submission and **will appear in the published article** if the submission is accepted. Please make sure it is accurate.<br><br>**Funded studies**<br>Enter a statement with the following details:<br>• Initials of the authors who received each award<br>• Grant numbers awarded to each author<br>• The full name of each funder<br>• URL of each funder website<br>• Did the sponsors or funders play any role in the study design, data collection and analysis, decision to publish, or preparation of the manuscript?<br><br>Did you receive funding for this work?"	
**Competing Interests**<br><br>Use the instructions below to enter a competing interest statement for this submission. On behalf of all authors, disclose any competing interests that could be perceived to bias this work—acknowledging all financial support and any other relevant financial or non-financial competing interests.<br><br>This statement is required for submission and **will appear in the published article** if the submission is accepted. Please make sure it is accurate and that any funding sources listed in your Funding Information later in the submission form are also declared in your Financial Disclosure statement.	The authors have declared that no competing interests exist.

Powered by Editorial Manager® and ProduXion Manager® from Aries Systems Corporation

View published research articles from *PLOS ONE* for specific examples.<br><br>**NO authors have competing interests**<br><br>Enter: *The authors have declared that no competing interests exist.*<br><br>**Authors with competing interests**<br><br>Enter competing interest details beginning with this statement:<br><br>*I have read the journal's policy and the authors of this manuscript have the following competing interests: [insert competing interests here]*<br><br>* typeset	
**Ethics Statement**<br><br>Enter an ethics statement for this submission. This statement is required if the study involved:<br><br>• Human participants<br>• Human specimens or tissue<br>• Vertebrate animals or cephalopods<br>• Vertebrate embryos or tissues<br>• Field research<br><br>Write "N/A" if the submission does not require an ethics statement.<br><br>General guidance is provided below. Consult the submission guidelines for detailed instructions. **Make sure that all information entered here is included in the Methods section of the manuscript.**	N/A

**Format for specific study types**<br><br>**Human Subject Research (involving human participants and/or tissue)**<br>• Give the name of the institutional review board or ethics committee that approved the study<br>• Include the approval number and/or a statement indicating approval of this research<br>• Indicate the form of consent obtained (written/oral) or the reason that consent was not obtained (e.g. the data were analyzed anonymously)<br><br>**Animal Research (involving vertebrate animals, embryos or tissues)**<br>• Provide the name of the Institutional Animal Care and Use Committee (IACUC) or other relevant ethics board that reviewed the study protocol, and indicate whether they approved this research or granted a formal waiver of ethical approval<br>• Include an approval number if one was obtained<br>• If the study involved *non-human primates*, add *additional details* about animal welfare and steps taken to ameliorate suffering<br>• If anesthesia, euthanasia, or any kind of animal sacrifice is part of the study, include briefly which substances and/or methods were applied<br><br>**Field Research**<br><br>Include the following details if this study involves the collection of plant, animal, or other materials from a natural setting:<br>• Field permit number<br>• Name of the institution or relevant body that granted permission	
**Data Availability**<br><br>Authors are required to make all data underlying the findings described fully available, without restriction, and from the time of publication. PLOS allows rare exceptions to address legal and ethical concerns. See the PLOS Data Policy and FAQ for detailed information.	No - some restrictions will apply

Powered by Editorial Manager® and ProduXion Manager® from Aries Systems Corporation

A Data Availability Statement describing where the data can be found is required at submission. Your answers to this question constitute the Data Availability Statement and **will be published in the article**, if accepted.<br><br>**Important:** Stating 'data available on request from the author' is not sufficient. If your data are only available upon request, select 'No' for the first question and explain your exceptional situation in the text box.<br><br>Do the authors confirm that all data underlying the findings described in their manuscript are fully available without restriction?	
**Describe where the data may be found in full sentences. If you are copying our sample text, replace any instances of XXX with the appropriate details.**<br><br>• If the data are **held or will be held in a public repository**, include URLs, accession numbers or DOIs. If this information will only be available after acceptance, indicate this by ticking the box below. For example: *All XXX files are available from the XXX database (accession number(s) XXX, XXX.).*<br>• If the data are all contained **within the manuscript and/or Supporting Information files**, enter the following: *All relevant data are within the manuscript and its Supporting Information files.*<br>• If neither of these applies but you are able to provide **details of access elsewhere**, with or without limitations, please do so. For example:<br><br>*Data cannot be shared publicly because of [XXX]. Data are available from the XXX Institutional Data Access / Ethics Committee (contact via XXX) for researchers who meet the criteria for access to confidential data.*<br><br>*The data underlying the results presented in the study are available from (include the name of the third party*	The data underlying the results presented in the study are available from Guangxi Key Laboratory of Functional Information Materials and Intelligent Information Processing. If accepted, we are committed to open-sourcing our research data and results.

and contact information or URL).<br>• This text is appropriate if the data are owned by a third party and authors do not have permission to share the data.<br><br>* typeset	
Additional data availability information:	

手稿 Click here to access/download;Manuscript;GR-AttNet.docx

GR-AttNet: Robotic Grasping with Lightweight Spatial Attention Mechanism

Shengbang Zhou [1,*,¶] **, Zhongsheng Liu** [1,¶] **, Chuanqi Li** [1] **, Shutian Liu** [2,3] **, Yadong Liu** [1]

[1] Guangxi Key Laboratory of Functional Information Materials and Intelligent Information Processing， Nanning Normal University, Nanning 530001 China;

[2] Guangxi Geographical Indication Crops Research Center of Big Data Mining and Experimental Engineering Technology, Nanning Normal University, Nanning 530001 China;

[3] Guangxi Key Laboratory of Earth Surface Processes and Intelligent Simulation, Nanning Normal University, Nanning 530001 China;

[*] Corresponding author Email: zhoushengbang@nnnu.edu.cn

[¶] These authors contributed equally to this work.

Funding: This research was supported by Guangxi Key Technologies R&D Program (No.AB241484046) and Guangxi Science and Technology Program (Project No. GUIKEAB23075177)

Abstract

Robotic grasping is crucial in manufacturing, logistics, and service robotics, but existing methods struggle with object occlusion and complex arrangements in cluttered scenes. We propose the Generative Residual Attention Network (GR-AttNet), based on the Generative Residual Convolutional Neural Network (GR-CNN). The spatial attention mechanism enhances its adaptability to clutter, and architectural optimization maintains high accuracy while reducing parameters and boosting efficiency. Experiments show GR-AttNet achieves 98.1% accuracy on the Cornell Grasping Dataset, 94.9% on the Jacquard Dataset, with processing speeds of 148 ms and 163 ms respectively, outperforming several state-of-the-art methods. With only 2.8 million parameters, it surpasses various existing models. Simulations further validate over 50% success rates across different scenario complexities. However, challenges remain in handling small object missed detection and severe occlusion cases. GR-AttNet offers a novel, highly practical solution for robotic grasping tasks.

Introduction

Robotic grasping is one of the core research topics in robotics and has long demonstrated extensive application value in multiple key areas such as industrial production, logistics transportation, and service robots. In recent years, with continuous technological advancements, robotic grasping is rapidly developing towards generalization and intelligence. For instance, home service robots need to flexibly grasp various items in complex environments to perform daily tasks. This trend has led to an increasing focus on how to improve the grasping performance of robots in complex environments, including adaptability to objects of different shapes, materials, and postures. The development of robotic grasping has a long history and can be classified into analytical methods and data-driven methods[Error! Reference source not found.]. Analytical methods primarily address grasping detection through the geometry, physical models, kinematics, or mechanics of objects. In contrast, data-driven methods imitate human grasping strategies to avoid complex mathematical and physical model computations. Data-driven methods can be further divided into grasping known objects and grasping unknown objects. For instance, Morales[2] and Miller[3] assume that the three-dimensional models of objects are available and generate optimal grasping poses by sampling the object surfaces. The method proposed by Fu[4] involves mimicking human grasping postures and iteratively improving the generated grasping poses through repeated trials. These are methods for grasping known objects. In contrast, Morrison[9] and Kumra[10] both utilize sensors to collect two-dimensional data of objects and generate candidate grasping poses based on heuristic algorithms, which are suitable for grasping unknown objects. Specifically, Morrison proposed the GG-CNN (Generative Grasping Convolutional

Neural Network), which predicts pixel-wise grasp configurations for a given depth image. Kumra introduced the GR-CNN (Generative Residual Convolutional Neural Network), which further improves grasp detection through residual learning.

As an advanced grasping detection network, GR-CNN has achieved remarkable results in multimodal input and efficient inference. However, its performance in complex environments still needs improvement, especially when handling background interference and small target grasping, where the model's adaptability is limited.In order to address the issues, we propose a new algorithm -- GR-AttNet (Generative Residual Attention Network). The main contributions of this paper can be summarized as follows:

1) Building upon GR-CNN, we propose GR-AttNet -- an architecturally optimized algorithm that addresses the prevalent issue where high-performance models typically require millions of parameters to support complex feature extraction and decision-making. Through network architecture refinement and integration of lightweight attention modules, GR-AttNet achieves significant performance improvements with 2.8M parameters. This design not only resolves low success rates in complex grasping scenarios but also mitigates the high computational burden of traditional models, maintaining high precision while enhancing real-time capability and deployment adaptability.

2) We propose a novel lightweight attention mechanism as one of GR-AttNet's core innovations. This mechanism automatically focuses on critical regions while suppressing irrelevant information in complex inputs. By integrating attention modules into GR-AttNet, the system achieves more precise target localization and significantly reduces interference from cluttered backgrounds during grasp detection.

Related Work

In the early days, researchers often relied on manual features. Saxena et al.(2008)[5] were the first to apply supervised learning to the robotic grasping detection task. By using the sliding window method and training a classifier with synthetic RGB images, they predicted grasping points. However, due to the lack of depth information, its adaptability in complex scenarios is limited. Subsequently, Jiang et al.(2011)[6] proposed an improved grasping detection method, introducing a seven- dimensional grasping box representation and fusing features through a multi-modal (RGB-D) deep network, significantly enhancing the accuracy and robustness of grasping detection. However, this method relies on a local search strategy to determine the optimal grasping pose, leading to high computational complexity and poor real-time performance.

With the development of deep learning, robotic grasping has also witnessed new opportunities. In the early stage of its development, the introduction of convolutional neural networks (CNN) significantly enhanced the feature extraction capabilities of the network. Lenz et al.(2015)[7] proposed a cascaded CNN framework, where the first stage generates candidate grasping boxes, and then a deep CNN selects the best pose. However, this method is time-consuming and has poor real-time performance, making it difficult to meet the requirements of rapid response in actual robot grasping tasks. Nevertheless, the cascaded CNN framework proposed by Lenz not only demonstrated the advantages of staged processing in improving detection accuracy but also provided ideas for further optimizing the network structure in subsequent research. Xia Jing et al.(2018)[8] combined the R-FCN (Region-based Fully Convolutional Network) and AngleNet modules. The method they proposed achieves significant breakthroughs in improving detection accuracy. However, the generalization ability of this method is limited in complex scenarios. Due to its reliance on specific training data and scene conditions, when faced with environments with complex backgrounds, varying lighting, or diverse object shapes, the model's adaptability is insufficient, restricting its application scope in practical scenarios. During the research process, researchers found that although the two-stage processing mechanism improves detection accuracy, it also increases computational complexity and processing time. This, to some extent, limits its application in scenarios with high real-time requirements.

To address the real-time performance deficiency of traditional two-stage networks, researchers have begun to explore single-stage methods. Among them, the GG-CNN proposed by Morrison et al.(2018)[9] is a significant milestone in the field of robotic grasping. This study was the first to apply the fully convolutional network (FCN) to grasping detection, directly generating grasping quality, angle, and width parameters through pixel-level regression, which significantly improved the speed and accuracy of grasping detection. GG-CNN adopts a fully convolutional network architecture, directly generating grasping parameters from the input depth image. This approach not only simplifies the computational flow but also enhances the efficiency and effectiveness of grasping detection. The inference time of GG-CNN is only 19ms, enabling a processing speed of over 50 frames per second, which meets the requirements of real-time closedloop control. On the Cornell Grasping dataset, the grasping success rate of GG-CNN achieved 73%, demonstrating its excellent performance.

In recent years, the research direction of grasping has gradually focused on integrating generative frameworks with lightweight residual structures. In 2020, Kumra et al.[10] proposed GR-CNN, a generative architecture that supports multi-modal (RGB-D) input. It achieves an inference time of 20 ms per frame. And on the Cornell Grasping dataset it attains an accuracy of 97.7% , with only 1.9 million parameters. Meanwhile, the development of attention mechanisms has been rapid, demonstrating strong performance across various fields. Some researchers have combined attention mechanisms with

generative frameworks to achieve better results. Lu et al.[11] embedded channel attention (SENet) and spatial pyramid in GR-CNN, further enhancing the grasping ability for small objects, with a speed of 23 ms per frame and an accuracy increase to 97.0%. Furthermore, the CBAM module[8], which integrates channel and spatial attention mechanisms, effectively suppresses background noise, increasing the detection accuracy for complex objects (e.g. wrenches) to 86%. These research achievements not only further improve the accuracy and efficiency of grasping detection but also provide better solutions for deployment in practical applications.

Methods

Grasp point definition

In this experiment, we consider the robot grasping task as predicting the poses of unknown objects from n-channel images. Unlike the seven-dimensional grasp representation used for spatial grasp, planar grasping typically employs a five-dimensional grasp representation. This experiment adopts a grasp representation method similar to that proposed by Morrison et al.[13]. Specifically, the grasping pose $\boldsymbol{G_r}$ in the robot coordinate system frame is represented as shown in (1).

$$G_r = (P, \Phi_r, W_r, Q) \tag{1}$$

In this experiment, we consider the robot grasping task as predicting the poses of unknown objects from n-channel images. Unlike the seven-dimensional grasp representation used for spatial grasp, planar grasping typically employs a five-dimensional grasp representation. This experiment adopts a grasp representation method similar to that proposed by Morrison et al..[13]. Specifically, the grasping pose G_r in the robot coordinate system frame is represented as shown in (1).

Where $P = (x, y, z)$ is the center position of the end-effector, Φ_r indicates the angle of rotation around the z-axis. W_r represents the opening width of the end-effector. And $Q$is the grasp quality score, which is used to evaluate the probability of success for the generated grasp pose. In detail, this score ranges from 0 to 1, with higher values corresponding to a greater probability of success.

Suppose we are conducting grasping detection on a n-channel image $I = \mathbb{R}^{n\times h\times w}$ with height w and width h. The grasping pose G_i generated in the image coordinate system is represented by(2).

$$G_i = (\bar{P}, \Phi_i, W_i, Q) \tag{2}$$

Where $\bar{P} = (x, y)$ presents the center point of the grasping in the image coordinate, Φ_i indicates the angle of rotation in the image coordinate system, which satisfies $\Phi_i \in [-\frac{\pi}{2}, \frac{\pi}{2}]$. W_i represents the width required for grasping, which satisfies $W_i \in [0, W_{max}]$ and Q is the scalar as in (1). It should be noted that W_{max} represents the maximum width that the end-effector can grasp.

To execute the grasping pose obtained in the image space on the robot, the following transformation is required to map the image coordinate system to the robot coordinate system, as shown in (3).

$$G_r = T_w^R[T_c^w(G_i)] \tag{3}$$

T_c^w is the transformation matrix that converts coordinates from the image coordinate system to the camera coordinate system based on the intrinsic parameters of the camera. Similarly, T_w^R maps the camera coordinate system to the robot coordinate system.

GR-AttNet architecture

GR-CNN achieves an end-to-end mapping from input n-channel images to grasping poses by integrating a generative architecture. This network can directly learn grasping poses from depth images without the need for pre-sampling of objects. Specifically, GR-CNN first crops the depth images into squares and then resizes them to a resolution of 224 × 224 pixels for input to the network. After passing through the GR-CNN network, it outputs a grasping box including the grasping center position, rotation angle, opening width, and grasping quality score. In this structure, less attention is paid to the background, which makes it difficult to accurately distinguish the target object from the background area.

In response to the above-mentioned problems, inspired by GR-CNN, we have designed a novel network architecture -- GR-AttNet. As shown in Fig 1, the proposed GR-AttNet model integrates n-channel images and depth map as the input. After passing through three convolutional layers, the generated feature maps are processed by the newly proposed attention mechanism module in this experiment and combined with the original feature maps. The combined feature maps are then input into three residual layers. To preserve the spatial features of the image and to ensure the accuracy of the coordinate transformations between the image coordinate system and the robot coordinate system, we up-sample the image to its original size by using a convolution transpose operation. Finally, two images are generated: one contains the grasping angle and width required by the robotic arm in the real world, and the other is used to evaluate the quality of the generated grasping box.

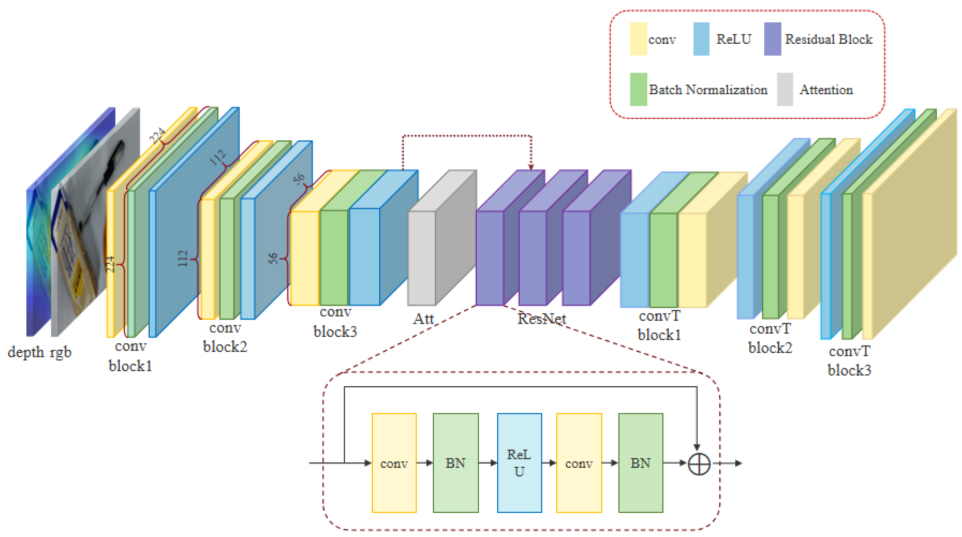

Fig 1. GR-AttNet architecture.

To ensure that the network can effectively perform feature extraction and processing, GR-CNN convolves the images into a size of 56×56, enabling the network to learn more abundant features while avoiding excessive computational load. In this experiment, we will continue to use this operation for image feature extraction. After convolution, the features are passed into the attention mechanism module that we proposed. The specific structure of this module will be detailed in the next section. Then, combined feature maps are fed into three residual networks, each of which consists of two convolutional layers, two normalization layers and an activation layer. The convolutional layer is responsible for feature extraction, the normalization layer is used to stabilize training and accelerate convergence, and the activation layer introduces non-linear characteristics to enhance the model's expressive power. This structural design is simple and efficient, capable of effectively processing input features and providing high-quality feature representations for subsequent tasks.

As we know, residual networks can effectively address the degradation problem in training deep neural networks, that is, the phenomenon where the training and validation errors increase with the increase of network depth. The residual network enables the network to learn the residual function $F(x) = H(x) - x$ instead of directly learning the objective function $H(x)$. This approach simplifies the optimization process. However, when the number of residual networks exceeds a certain limit, it can lead to problems such as vanishing gradients and dimensional errors, resulting in a decrease in accuracy. Given that the network achieves effective feature fusion through our attention mechanism, the residual block can fully capture image

features with only a three-layer structure. Therefore, to reduce the number of model parameters, the residual network in this experiment is designed to have three layers.

The network proposed in this experiment only contains 2.8 million parameters. Compared with other networks mentioned in references[14][15], its structure is more concise. Therefore, when compared to similar grasping prediction techniques that utilize complex architectures and involve millions of parameters, this network demonstrates significant advantages. It achieves lower computational costs and faster running speeds, making it more computationally efficient overall.

Attention block

The spatial attention mechanism assigns varying weights to different positions within the input image based on their significance, thereby enhancing the model's focus on and extraction of key information[16]. Additionally, the attention mechanism adaptively adjusts the weights of channels, which strengthens GR-AttNet's ability to learn grasping-related features. This adaptive weighting effectively suppresses irrelevant feature information, reduces prediction bias, and consequently improves the model's robustness and the accuracy of grasping detection.

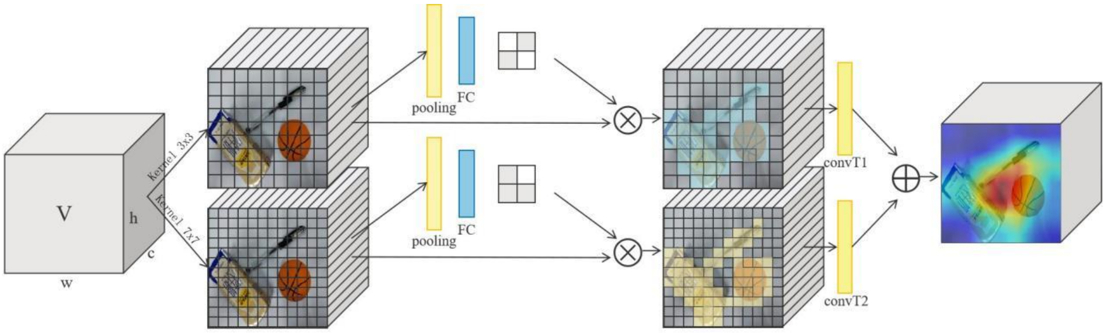

Fig 2. Structure of attention mechanism block.

Inspired by the structure of SK-Net[17], this experiment designs the attention mechanism module as shown in Fig 2. This module introduces multiple parallel convolutional kernel branches with different receptive fields to learn the weights of feature maps at different scales, enabling the GR-AttNet network to select more appropriate multi-scale feature representations. Assuming the input feature map V has dimensions $h \times w \times c$, where h and w are the spatial height and width of the feature map, and c is the number of channels. The specific representation is given by the following (4).

$$V = [v^{(1,1)}, v^{(1,2)}, \ldots, v^{(i,j)}, \ldots, v^{(h,w)}] \quad (4)$$

Where $v^{(i,j)} \in \mathbb{R}^{(1\times1\times c)}$, $i \in [1,h]$, $j \in [1,w]$. The input feature map V is first convolved with kernels of sizes *3×3* and 7×*7*. The selection of these two sizes of convolution kernels will be discussed in detail in Conclusion. The smaller kernel *(3×3)* is suitable for extracting detailed information, while the larger kernel *(7×7)* covers a broader receptive field to capture more extensive contextual information. To facilitate subsequent calculations, this study employs the "same" padding method, ensuring that the size of the feature map after convolution remains consistent with the original feature map. The specific convolution processes are denoted as $U = K_{sq} * V$ and $U' = K'_{sq} * V$, where $K_{sq} \in \mathbb{R}^{(3\times3\times c)}$, $K'_{sq} \in \mathbb{R}^{(7\times7\times c)}$, $U \in \mathbb{R}^{(H,W)}$, $U' \in \mathbb{R}^{(H,W)}$. Afterwards, average pooling is applied to each channel of the two convolved feature maps, resulting in the global spatial features for each channel, forming two tensors of size $h \times w \times 1$. Channel-wise average pooling effectively captures the global information of each channel without increasing the model's parameter count. Subsequently, the pooled feature maps are passed through a fully connected layer that includes a Sigmoid function, which performs a nonlinear transformation on the feature vectors. The Sigmoid function normalizes the attention scores into a probability distribution, ensuring that the attention weights for each spatial location fall between 0 and 1. This process yields the weights for each spatial position.

Then, the generated weights are multiplied element-wise with the original feature map. As shown in (5) and (6), this adjusts the contributions of each channel in the original feature map, enabling the model to focus more on important features while suppressing less important ones.

$$\hat{V} = [\sigma(u_{(1,1)})v^{(1,1)}, \sigma(u_{(1,2)})v^{(1,2)}, \ldots, \sigma(u_{(i,j)})v^{(i,j)}, \ldots, \sigma(u_{(h,w)})v^{(h,w)}] \quad (5)$$

$$\hat{V}' = [\sigma(u'_{(1,1)})v'^{(1,1)}, \sigma(u'_{(1,2)})v'^{(1,2)}, \ldots, \sigma(u'_{(i,j)})v'^{(i,j)}, \ldots, \sigma(u'_{(h,w)})v'^{(h,w)}] \quad (6)$$

Finally, the two feature maps $\hat{V}$ and $\hat{V}'$ are fused at the pixel level as shown in (7).

$$\tilde{V} = \hat{V}' + \hat{V} \quad (7)$$

The attention mechanism learns the importance of different channels in the input feature map and adjusts it accordingly. thereby enhancing the model's ability to capture key information. All in all, the introduction of the attention mechanism enhances the model's ability to capture key information.

Training and Results

Evaluation standards

To objectively compare the performance of different grasping detection models, this study introduces the evaluation standard proposed by Jiang et al.[6] in 2011 to evaluate the accuracy of the grasping box. According to this rectangular metric, a grasping prediction is deemed correct if it meets the following conditions.

1) The difference between the predicted grasping angle and the actual grasping angle is within 30° .

2) The Jaccard index between the predicted grasping rectangle and the actual grasping rectangle is greater than 0.25. The definition of the Jaccard index is as follows:

$$J(G_P, G_t) = \left|\frac{G_P \cap G_t}{G_P \cup G_t}\right| > 0.25 \tag{8}$$

Where G_P represents the predicted capture rectangle, G_t represents the true grasping rectangle.

Grasp datasets

In the field of robotic planar grasping, the number of publicly available datasets is limited. Table 1 summarizes several representative datasets, each with its own merits and demerits. The Cornell Grasping dataset is the most common benchmark dataset, containing 1,035 RGB-D images and 8,019 grasping annotations, covering 240 objects. However, its scenes are relatively simple, with only a single object in each image, making it difficult to meet the grasping requirements in complex scenarios. The Jacquard dataset is larger in scale, with 54,000 RGB-D images and 1.1 million grasping annotations, covering 11,619 objects. Its synthetic data approach provides rich diversity, but there is a certain gap from real-world scenarios. Grasp-Net further expands the scale and complexity, with 97,000 RGB-D images and 1.2 billion grasping annotations, covering 88 objects, and supports multi-object scenarios (1-10 objects) and 6-DOF grasping pose annotations. However, the processing and training costs of large-scale data are relatively high. DexNet focuses on depth images, containing 6.7 million depth images and 6.7 million grasping annotations, covering 1,500 objects. Its synthetic data is helpful for studying the robustness of grasping algorithms, but it only relies on depth information and may be limited in complex visual scenarios. The VR-Grasping-101 dataset is generated through virtual reality technology, containing 10,000 RGB-D images and 4.8 million grasping annotations, covering 101 objects. The Multi-object dataset is based on the annotation method of the Cornell Grasping dataset, placing multiple objects in the same single scene for recognition, containing 96 RGB-D images and 2,904 grasping annotations. It makes up for the deficiency of the Cornell Grasping dataset in complex scenarios and provides an experimental basis for multi-object grasping research.

Table 1. Different types Datasets.

Datasets Name	Images	Grasps	Source	Scene	Method
**Cornell[7]**	1,035	8,019	Real	Single object	grasping box
**Jacquard[18]**	54,000	1.1M	Synthesis	Single object	grasping box
**Grasp-Net[19]**	97,000	1.2B	Real	Multiple objects (1~10)	6-DOF pose
**Dex-Net[20]**	6.7M	6.7M	Synthesis	Single object	grasping box
**VR-Grasping-101[21]**	10,000	4.8M	Synthesis	Single object	6-DOF pose
**Multi-Object[22]**	96	2,904	Real	Multiple objects (4~5)	grasping box

Firstly, we focus on the testing of individual objects. To ensure that the objects closely resemble real-world scenarios, the Cornell and Jacquard datasets were selected as experimental subjects. Additionally, to demonstrate the method's superiority in cluttered environments, the Multi-Object dataset was also tested. These three datasets contain objects of different shapes, sizes, materials, and textures, which can comprehensively evaluate the performance of the network under various conditions.

Training results

To thoroughly investigate the impact of kernel size in the proposed attention module on network performance, a series of experiments were designed in this paper. By selecting convolutional kernels of different sizes and applying them to the constructed network architecture, detailed records and analyses were conducted during the training and testing processes of the network. The experimental results are shown in Table 2, which indicate that changes in kernel size have a significant influence on the performance of the network structure. Convolutional kernels of different sizes exhibit varying performance in terms of feature extraction, computational complexity, and model generalization ability. Specifically, in the network architecture proposed in this paper, convolutional kernels of size 3×3 and 7×7 achieved the highest accuracy, reaching 98.1% on the Cornell dataset, with only 2.8M parameters. On the Jacquard dataset, convolutional kernels of size 5×5 and 7×7 achieved the highest accuracy of 95.1%, but the number of parameters also increased to 3.1M. Taking all factors into consideration, convolutional kernels of size 3×3 and 7×7 were selected for subsequent experiments.

Table 2. The effect of kernel size on the experiment

Size			Parameters	Accuracy(%)	
3*3	5*5	7*7		Cornell	Jacquard

√			2.0M	0.924	0.908
	√		2.3M	0.945	0.913
		√	2.7M	0.955	0.929
√	√		2.4M	0.954	0.942
√		√	2.8M	0.981	0.949
	√	√	3.1M	0.963	0.951
√	√	√	3.3M	0.967	0.943

Due to the relatively small size of the Cornell dataset, five-fold cross-validation is adopted in this paper. Table 3 shows the accuracy rates of different grasping detection models on the Cornell dataset. The GR-AttNet achieves an accuracy rate of 98.1%, which is higher than the benchmark GR-CNN's 97.7%. Moreover, compared with other large-scale networks, GR-AttNet has only 2.8M parameters, making it more deployable. Table 4 presents the accuracy rates of various models on the Jacquard dataset. GR-AttNet achieves an accuracy rate of 94.9%, which is also higher than the benchmark GR-CNN's 94.6%. Although the baseline method is theoretically capable of achieving an inference speed of 20 ms, we found that its actual running speed is about 200 ms in our tests. In contrast, the method proposed in this paper only takes 148 ms and 163 ms to complete inference in practical testing.

Table 3. Performance of different models on cornell.

Author	Network structure	Accuracy(%)	Parameters	Speed(ms)
**Jiang[6]**	**Fast Search**	60.5	\	5000
**Morrison[9]**	**GG-CNN**	73	62000	19
**Lenz[7]**	**SAE**	73.9	\	1350
**Redmon[23]**	**AlexNet**	88	~6.1M	76
**Kumra[24]**	**GR-CNN**	97.7	1.9M	20
**Lou[25]**	**GR-CNN+CBAM**	\	6.1M	\
**ours**	**GR-AttNet**	98.1	2.8M	148

Table 4. Performance of different models on jacquard

Author	Network structure	Accuracy(%)	Speed(ms)
**Depierre[18]**	**Jacquard**	74.2	\
**Morrison[9]**	**GG-CNN2**	84	20
**Zhou[14]**	**FCGN**	91.8	117
**Kumra[24]**	**GR-CNN**	94.6	20
**Yu[26]**	**PTGNet**	94.8	42.5
**ours**	**GR-AttNet**	94.9	163

In summary, GR-AttNet can perform the planar grasping task well. However, due to the introduction of the attention mechanism, although the model has fewer parameters, it leads to a more complex computational process compared to GR-CNN, requiring more computing resources for processing. Therefore, GR-AttNet is slightly inferior to other highperformance models in terms of inference speed.

To present the research results of this paper more intuitively, the visualization results of the grasping outcomes are shown in Fig 3. RGB represents the original RGB image. Att displays the image effect after processing by the designed attention mechanism module. The target area is effectively highlighted. It is evident from this that the module can precisely focus on the items to be grasped. Grasp is the grasping box generated by the network, accurately marking the grasping position. Q represents the final grasping quality map, which visually reflects the reliability of the grasping operation. Moreover, we tested the proposed method on the Multi-Cornell dataset and achieved an accuracy of 89.2%, with some of the grasping results shown in Fig 4

These visualized results clearly demonstrate that GR-AttNet is capable of accurately generating grasping boxes, thereby providing precise guidance for subsequent grasping operations.

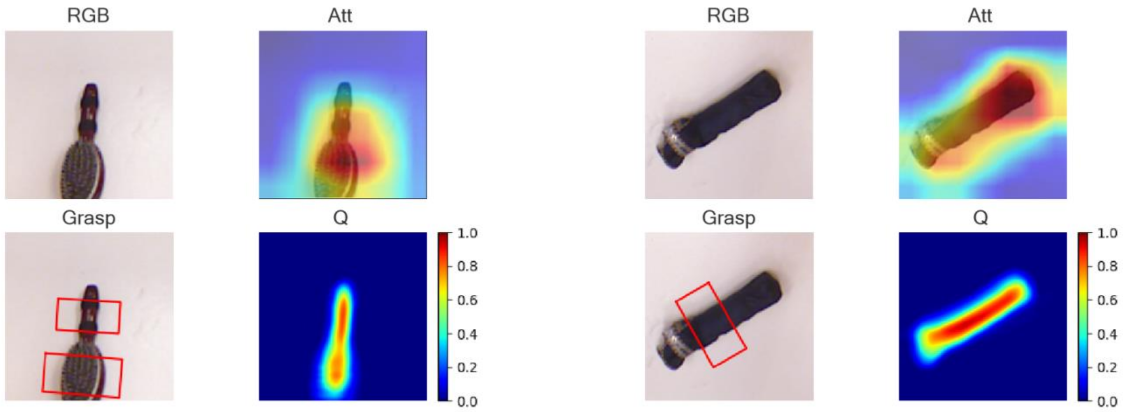

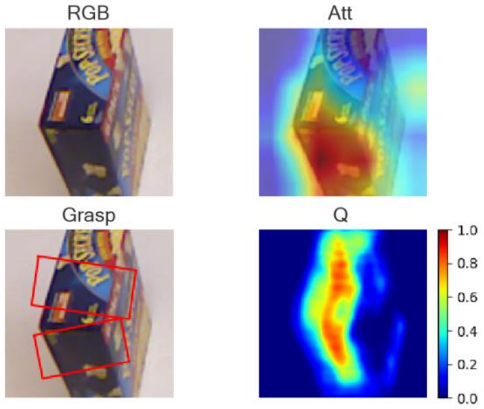

Fig 3. Visualization of the single results: RGB represents the original RGB image; Att displays the image effect after processing by the designed attention mechanism module; Grasp is the grasping box generated by the network; Q represents the final grasping quality map.

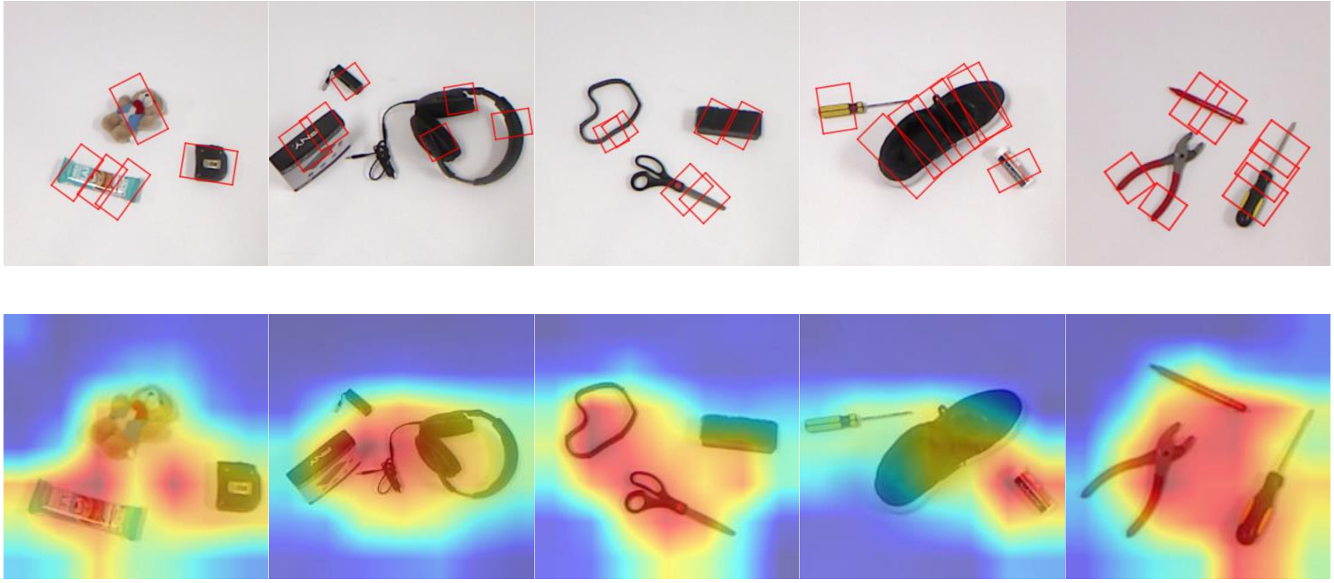

Fig 4. Visualization of the Multi-Objects results. The upper image shows the grasping boxes generated by the GR-Att network, with multiple boxes for each object. The most feasible one is selected based on the position of each box. The lower image shows the attention regions generated by the Att module, which can be seen to cover all objects well.

Simulation Verification

Simulation environment setup

To reduce costs, the simulation in this experiment was conducted using PyBullet in a Windows environment. PyBullet is an open-source Python module based on the Bullet physics engine, widely used in robotics simulation, reinforcement learning, game development, and computer graphics research. Complex physical phenomena (e.g. collision detection, friction) can be effectively simulated through PyBullet's rigid-body dynamics engine, which also exhibits high computational efficiency in real-time scenarios.

The robotic arm model selected is the UR5, which features six rotary joints with motion ranges of $[0, 2\pi]$ for each joint. Its end-effector is a two-finger gripper. The UR5 is characterized by high precision and sensitivity, and it offers high control efficiency. The comprehensive ecosystem of the UR5 enables it to be easily integrated with other technologies to achieve object detection and complex grasping tasks. The simulation process is shown in Fig 5 and mainly consists of the following four steps:

1) Environment Setup: In PyBullet, we construct a simulation environment that includes a robotic arm and a variety of objects. We set all objects as rigid bodies, including many irregular objects such as keychains and dolls. We randomly place these objects on the ground, while the position of the robotic arm is fixed.

2) Visual Perception: Depth pixel information of target objects was acquired through an RGB-D camera (Fig 6).

3) Grasp Prediction: Object grasp poses were predicted using the GR-AttNet network.

4) Execution & Evaluation: The best predicted grasping box is mapped to the simulated robotic arm to perform the grasp. If an object is successfully grasped, it is removed from the environment. A maximum of 5 grasp attempts were allocated per object. If all attempts failed, the system reinitialized visual perception and generated new grasp proposals (returning to Step 2).

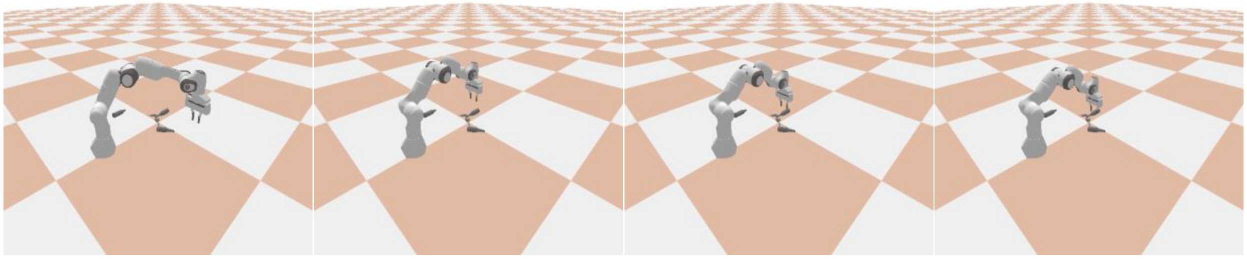

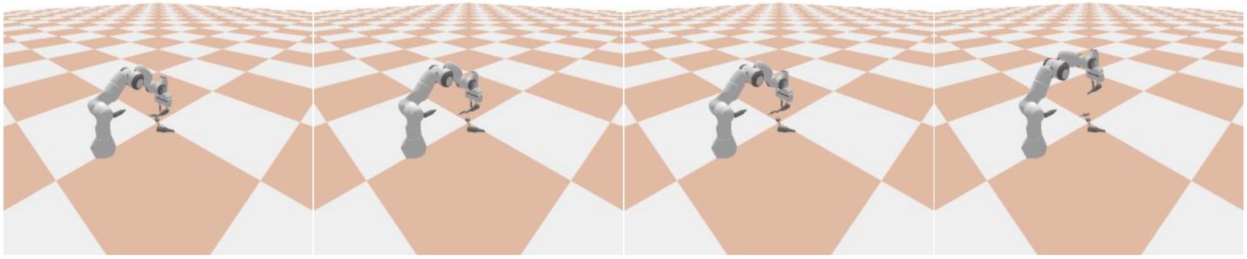

Fig 5. Simulation environment and specific capture process. Starting from the first image on the left in the first row and moving to the right, it illustrates a specific grasping process step by step.

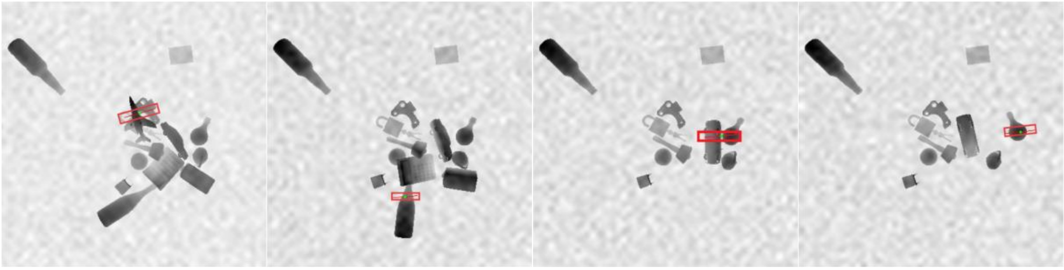

Fig 6. Visual Perception. The camera captures information and generates a grasping box for one object at a time. After the robotic arm successfully grasps the object, the successfully grasped object is deleted from the environment, and the process continues in this manner.

Table 5. Simulation and grasping results of different kinds of objects.

Species	Total number	Number of success	Failure times			Accuracy(%)
			slippage times	Occlusion errors	Recognition errors	
1	59	47	2	0	10	79.70%
5	318	247	18	10	43	77.67%
10	611	412	52	76	71	67.43%
15	1639	701	152	282	504	42.77%

As evidenced by the data in Table 5, the grasping success rate reaches a relatively high level of 79.7% in single-object scenarios. The term "slippage times" refers to the number of failures during the grasping process where the gripper comes into

contact with other objects, leading to inaccurate recognition and subsequent failure. "Occlusion errors" refer to the number of failures where the relationship between objects in terms of depth is not correctly identified. As a result, the generated grasping box directs the robotic arm to grasp an object that is further back first, leading to failure. "Recognition errors" refer to the number of failures caused by the generation of unreasonable grasping boxes during the grasping process.

However, a progressive decline in success rate is observed with increasing object quantity, where the rate of deterioration accelerates proportionally with scene complexity. Through simulation visualization and further analysis, the following critical issues have been identified.

For small-sized objects, although the network can accurately identify their features and generate corresponding grasping poses, the physical grasping process often fails due to object slippage caused by their miniature dimensions. Conversely, for large-sized objects, the network occasionally produces invalid grasping boxes that exceed the maximum grasping range, similarly resulting in failed grasping operations.

Furthermore, the network demonstrates significant limitations in handling occlusions between objects. When the network initially identifies a partially occluded object and generates a grasp pose, the end-effector frequently collides with obstructing objects during approach. This unintended contact displaces the target object, ultimately causing grasp failure.

Conclusion

This study proposes a multi-modal end-to-end grasping framework -- GR-AttNet. By introducing the attention mechanism and optimizing the network architecture, the system significantly improves grasping detection accuracy and robustness in cluttered scenarios.

The experimental results show that GR-AttNet achieves an accuracy of 98.1% and 94.9% on the Cornell Grasping dataset and the Jacquard dataset. The network shows good adaptability in the grasping task of different numbers of objects in the simulated environment, and still achieves a 50% grasping success rate in the face of severe occlusion. However, sometimes there is still the problem of not obtaining the target position in real time, and in the face of antagonistic objects, the network processing results and the actual situation are quite different. Despite these limitations, the proposed GR-AttNet demonstrates

robust generalization capabilities and practical feasibility, establishing a valuable reference framework for robotic planar grasping applications in real-world environments.

Acknowledgments

This research was supported by Guangxi Key Technologies R&D Program (No.AB241484046) and Guangxi Science and Technology Program (Project No. GUIKEAB23075177). We also gratefully acknowledge the Guangxi Key Laboratory of Functional Information Materials and Intelligent Information Processing for providing experimental facilities and technical support.

References

8/26

[1] Bohg J, Morales A, Asfour T, et al. Data-driven grasp synthesis -- A survey[J]. IEEE Transactions on Robotics, 2014, 30(2): 289-309.

[2] Morales A, Asfour T, Azad P, et al. Integrated grasp planning and visual object localization for a humanoid robot with five-fingered hands[C]. IEEE/RSJ International Conference on Intelligent Robots and Systems. Beijing: IEEE, 2006: 5663-5668

[3] Miller A T, Knoop S, Christensen H I, et al. Automatic grasp planning using shape primitives[C]. Proceedings IEEE International Conference on Robotics and Automation. Taipei: IEEE, 2003: 1824-1829.

[4] Fu, Zipeng, Tony Z. Zhao, and Chelsea Finn. “Mobile ALOHA: Learning Bimanual Mobile Manipulation with Low-Cost Whole-Body Teleoperation.” Conference on Robot Learning (CoRL), 2024.

[5] Saxena A, et al. Learning grasps for objects. In: Proceedings of the IEEE International Conference on Robotics and Automation. 2008.

[6] Jiang Y, Moseson S, Saxena A. Efficient grasping from RGBD images: Learning using a new rectangle representation[C]. IEEE International Conference on Robotics and Automation. Shanghai: IEEE, 2011.

[7] Lenz I, Lee H, Saxena A. Deep learning for detecting robotic grasps[J]. International Journal of Robotics Research, 2015, 34(4-5): 705-724.

[8] Xia J., Qian K., Ma X.-D., et al., "Fast detection of planar grasp pose for robots based on cascaded convolutional neural network," Robot, vol. 40, no. 6, pp. 794-802, 2018.

[9] Morrison D, Corke P, Leitner J. The grasp generation convolutional neural network[J]. arXiv preprint arXiv:1803.01619, 2018.

[10] Kumra S, Kanan C. Robotic grasp detection using deep convolutional neural networks[J]. IEEE Robotics and

Automation Letters, 2020.

[11] Lu Z.-L., Lin W., Zeng B., et al., "Real-time detection method for robotic target grasping regions," Computer Engineering and Applications, no. 9/10/11/12, pp. 1-10, 2019.

[12] Woo S, Park J, Lee J Y, et al. CBAM: Convolutional Block Attention Module [C]// Proceedings of the European Conference on Computer Vision (ECCV). Munich: Springer, 2018: 3−19.

[13] Morrison D, Corke P, Leitner J. Learning robust, real-time, reactive robotic grasping[J]. The International Journal of Robotics Research, 2020, 39(2-3): 183-201.

[14] Zhou X, Lan X, Zhang H, Tian Z, Zhang Y, Zheng N. Fully convolutional grasp detection network with oriented anchor box[C]. In: 2018 IEEE/RSJ International Conference on Intelligent Robots and Systems (IROS). IEEE, 2018: 7223-7230.

[15] Ogas E, Avila L, Larregay G, Moran D. A robotic grasping method using convnets[C]. In: 2019 Argentine Conference on Electronics (CAE). IEEE, 2019: 21-26.

[16] Roy, Abhijit Guha, Nassir Navab, and Christian Wachinger. "Concurrent Spatial and Channel 'Squeeze & Excitation'in Fully Convolutional Networks." arXiv preprint arXiv:1803.02579 (2018).

[17] Cai B, Liu M, Lu Z, et al. CK-ATTnet: Medical image segmentation network based on convolutional kernel attention[J]. Computers in Biology and Medicine, 2024, 171: 106785.

[18] Depierre A, Dellandrea E, Chen L. Jacquard: A large-scale dataset for robotic grasp detection [C]. In: 2018 IEEE/RSJ International Conference on Intelligent Robots and Systems (IROS). Madrid, Spain, Oct. 1-5, 2018.

[19] Fang B, Liu Y, Li J, et al. GraspNet-1Billion: A large-scale benchmark for general object grasping[J]. IEEE Robotics and Automation Letters, 2020, 5(2): 1535-1542.

[20] Mahler J, Liang P, Niyogi S, et al. Dex-Net 3.0: Computing robust robot grasps with a generative model[J]. IEEE Robotics and Automation Letters, 2018, 3(4): 3355-3362.

[21] Yan X, Khansari M, Bai Y, Hsu J, Pathak A, Gupta A, Davidson J, Lee H. Learning to predict grasping interaction with geometry-aware 3D representations[J]. 2018 IEEE International Conference on Robotics and Automation (ICRA), 2018: 1-8.

[22] Fu-Jen Chu, Ruinian Xu, Patricio A. Vela. Real-world Multi-object, Multi-grasp Detection[J]. IEEE Robotics and Automation Letters, 2018, 3(4): 3355-3362.

[23] Redmon, Joseph, and Anelia Angelova. "Real-Time Grasp Detection Using Convolutional Neural Networks." 2015 IEEE International Conference on Robotics and Automation (ICRA) (2015): 1316-1322.

[24] Kumra, Shuran, Anirudh Agarwal, and Ashutosh Saxena. "Antipodal Robotic Grasping Using Generative Residual Convolutional Neural Network." 2020 IEEE/RSJ International Conference on Intelligent Robots and Systems (IROS) (2020): 1140-1147.

[25] Lou X.-H., Mao P.-J., Niu X.-Y., Duan Y.-T., "An improved grasp detection algorithm based on GR-CNN and its simulation," Modeling and Simulation, vol. 12, no. 6, pp. 5196-5206, 2023. DOI: 10.12677/mos.2023.126473.

[26] Yu Q.-S., Xu X.-R., Liu Y.-Z., "Transformer-based pixel-level robotic grasp pose detection," Journal of Engineering Design, vol. 31, no. 2, pp. 238-247, 2024. DOI: 10.3785/j.issn.1006-754X.2024.03.150.

Supporting information

List Supporting Information captions at the end of the manuscript in a section titled "Supporting information". Supporting Information files do not require full captions; only labels ("S1 Fig") are fully required.
