# Supplementary material for: GR-AttNet: Robotic grasping with lightweight spatial attention mechanism
Source: PLoS One. 2025 Dec 4;20(12):e0337599. doi: 10.1371/journal.pone.0337599 (PMC12677535; doi:10.1371/journal.pone.0337599)
Supplement: Attachment — Submitted filename: Response to Reviewers.docx [file pone.0337599.s003.docx]

**RESPONSE TO THE EDITOR’S AND REVIEWERS’ COMMENTS**

GR-AttNet: Robotic Grasping with Lightweight Spatial Attention Mechanism

(Manuscript Number: PONE-D-25-28907)

Dear Academic Editor and Reviewers,

Thank you for your leter and for the reviewers’ comments concerning our manuscript. Those comments are all valuable and very helpful for revising and improving our paper, as well as the important guiding significance to our researches. We have carefully revised our manuscript based on the comments and suggestions, and addressed all the concerns of the editors and reviewers in the updated manuscript, with changes aimed at improving clarity highlighted in yellow or shown in blue text accordingly.

Sincerely yours

Shengbang Zhou

**Academic Editor**:

Many thanks for your time and efforts in reviewing our manuscript. Your insightful comments have helped us significantly improve the quality of this paper. Our detailed reflections on your comments are provided below point by point.

**1.**Improvements of the results compared with existing works should be clearly presented and discussed.

**The author’s answer:**

- Thank you very much for your comments. We have revised the manuscript to more clearly present and discuss the improvements of our method compared to existing works, focusing on accuracy, model efficiency, and adaptability in cluttered scenes. Below is a summary of key improvements, with references to the highlighted sections in the revised manuscript.
- Firstly, we have enriched the **Related Work** section by providing a detailed analysis of the limitations inherent in existing grasping algorithms, and by clearly highlighting the advantages of our proposed GR-AttNet model (**See Lines 110–118 and 121–138 on Pages 6–7**). The proposed method effectively balances real-time performance and accuracy. By integrating a lightweight attention mechanism, GR-AttNet demonstrates superior capability in handling complex scenarios, while maintaining inference speeds of approximately 150 milliseconds, thus better satisfying the demands of real-time robotic applications.
- Additionally, we have conducted statistical significance tests to validate the observed improvements in accuracy, with detailed results presented **in Table 2** (**Lines 375–385, highlighted in blue**).

**2.**References can be further enhanced while the writing of whole manuscript is suggested to improve.

**The author’s answer:**

- Thank you very much for your comments. We have added **14 recently published high-quality papers** to better reflect the latest progress in robotic grasping and attention-based methods. All newly cited works now appear in the Related Work and Introduction sections, and every reference has been double-checked for completeness and format consistency **(See the updated Reference list, highlighted in yellow)**.
- The entire manuscript has been carefully revised for grammar, clarity and readability. We benefited from a professional English-language editing service to polish the text, ensuring that the arguments flow logically and conform to academic standards. Major linguistic improvements can be found in the Abstract, Results and Discussion, and Conclusion sections.
- We sincerely thank you for the insightful suggestions, all of which have been carefully addressed in the revised manuscript. We believe these revisions noticeably raise the overall quality of the paper.

**Editorial Requirement**:

Thank you for pointing out the issues in the submission process. I will address them one by one and make the necessary revisions according to the requirements when resubmitting.

**1.**Please ensure that your manuscript meets PLOS ONE's style requirements, including those for file naming.

**The author’s answer:**

- Thank you for pointing out this issue. We have downloaded the template from the official website and modified the manuscript format.

**2.** Please note that PLOS One has specific guidelines on code sharing for submissions in which author-generated code underpins the findings in the manuscript.

**The author’s answer:**

- We thank the editor for highlighting PLOS ONE’s code-sharing policy. In compliance with the journal’s requirement that “all author-generated code must be made available without restriction upon publication,” we have deposited the complete, reproducible code in a public repository (Zenodo) and obtained a DOI.
- **DOI: 10.5281/zenodo.17356618**
- **Repository: https://github.com/zsliu0304/GR-AttNet**
- **License: MIT**
- The Data Availability Statement in the manuscript has been updated accordingly.

**3.** We note that the grant information you provided in the ‘Funding Information’ and ‘Financial Disclosure’ sections do not match.

**The author’s answer:**

- We thank the editor for pointing out the oversight. Our study was financially supported by two grants from Guangxi, China:
- Guangxi Key Technologies R&D Program (No.AB241484046)
- Guangxi Science and Technology Program (Project No. GUIKEAB23075177)

**4.** Please state what role the funders took in the study.

**The author’s answer:**

- We thank the editor for pointing out the oversight. The funders had no role in study design, data collection and analysis, decision to publish, or preparation of the manuscript.

**5.** Thank you for stating the following in the Acknowledgments Section of your manuscript.....

**The author’s answer:**

- We have removed all funding-related sentences from the Acknowledgments section and any other parts of the manuscript. The complete, amended Funding Statement is as follows:
- "This research was funded by the Guangxi Key Technologies R&D Program (Grant No. AB241484046) and the Guangxi Science and Technology Program (Grant No. GUIKEAB23075177)."
- The Guangxi Key Laboratory of Functional Information Materials and Intelligent Information Processing is our institutional affiliation and provided experimental facilities and technical support, but it did not provide direct financial support; therefore it is not listed in the Funding Statement.

**6.** In the online submission form, you indicated that your data is available only on request from a third party. Please note that your Data Availability Statement is currently missing the contact details for the third party, such as an email address or a link to where data requests can be made. Please update your statement with the missing information.

**The author’s answer:**

- Thank you for your reminder. We have updated the Data Availability Statement to include the missing third-party contact details:
- "All image datasets used in this study are publicly available from the Cornell and Jacquard repositories. The Cornell Grasping Dataset can be requested via the contact form at http://pr.cs.cornell.edu/grasping/rect_data/data.php.
- The Jacquard Dataset is available at https://jacquard.liris.cnrs.fr/ after completing the online access form."
- We have entered these e-mail addresses and URLs in the online submission form on the "Data Availability" page.

**7.** Please ensure that you have an ORCID iD and that it is validated in Editorial Manager.

**The author’s answer:**

- Thank you for your reminder. We have created and validated the ORCID iD for the corresponding author in Editorial Manager.
- ORCID iD: 0009-0005-7625-1935
- The link has been authenticated via the "Fetch/Validate" button under "Update my Information" and now appears as verified in the system.

**8.** Please upload a copy of S1 Figure, to which you refer in your text on page 20. If the figure is no longer to be included as part of the submission please remove all reference to it within the text.

**The author’s answer:**

- Thank you for your reminder. The template text referring to “S1 Figure” on page 20 has been removed in the revised manuscript; no supplementary figure is supplied and all related in-text references have been deleted. We apologise for the oversight.

**9.** If the reviewer comments include a recommendation to cite specific previously published works, please review and evaluate these publications to determine whether they are relevant and should be cited. There is no requirement to cite these works unless the editor has indicated otherwise.

**The author’s answer:**

- We have carefully reviewed the reviewer comments and confirm that no specific previously published works were recommended for citation. However, to further strengthen the discussion, we have added several new references (highlighted in the text).

**Reviewer #1**:

Many thanks for your time and efforts in reviewing our manuscript. Your insightful comments have helped us significantly improve the quality of this paper. Our detailed reflections on your comments are provided below point by point.

1. However, no statistical significance testing for accuracy improvements, limited discussion on why 3*3 or 7*7 kernels outperformed other combinations, and simulation testing only in PyBullet without real-world validation.

**The author’s answer:**

- Thank you very much for your thorough review and invaluable comments. In response to your three major concerns
- **(1) There is no statistical significance testing for the accuracy improvements.**
- We sincerely agree with you that statistical significance testing is crucial for validating the reliability of performance improvements. In response to this comment, we have now incorporated a more rigorous statistical analysis into our revised manuscript. Specifically, we have:

I) Clearly stated the use of five-fold cross-validation in our experiments.
II) Reported the mean and standard deviation from multiple independent runs **in Table 2** (**e.g., achieving 98.1% ± 0.2% on the Cornell Dataset**).

- **(2) Limited discussion on why 3x3 or 7x7 kernels outperformed other combinations.**
- We thank the reviewer for this insightful observation. We have now expanded our discussion **in Section 4.1 "Attention mechanism results"** to provide a theoretical rationale for the superior performance of the 3*3 and 7*7 kernel combination. The key reasons are as follows:

I) The **3*3 convolutional kernel** is highly effective at capturing fine-grained local details (such as edges and textures), which is essential for the precise localization of grasp points.
II) The **7*7 convolutional kernel**, with its larger receptive field, excels at extracting broader contextual information (such as object contours and spatial relationships within a cluttered scene).

The combination of these two kernels allows our model to integrate multi-scale features effectively, achieving an optimal balance between local precision and global context awareness, which is critical for grasp detection in complex environments.

- This explanation is now supported by the comparative results of various kernel combinations presented **in Table 2**.
- **(3) Simulation testing was only conducted in PyBullet without real-world validation.**
- We fully recognize that real-world validation is pivotal to robotic grasping research. Owing to hardware constraints, however, we are presently unable to conduct physical trials on a UR5 or similar manipulator. PyBullet was selected because it is a high-fidelity, widely adopted physics engine that accurately reproduces cluttered scenes with heavy occlusions and up to fifteen interacting objects. We nevertheless acknowledge that a pure-simulation evaluation inevitably limits direct transferability to the real world.
- To strengthen the credibility of our findings, the revised manuscript includes extensive ablation studies and employs **Wilson score** intervals to quantify the statistical uncertainty of every reported success rate. As detailed in the discussion below **Table 5**, single-object grasping achieves **79.7 %** success with a 95 % CI of [68.1 %, 87.8 %], indicating stable performance. As scene complexity increases, the intervals widen markedly: 95 % CIs for 5, 10 and 15 objects are [72.8 %, 81.7 %], [63.6 %, 71.0 %] and [40.4 %, 45.2 %], respectively. In contrast to prevailing methods that report only single-object accuracy, GR-AttNet delivers statistically meaningful confidence bounds under severe occlusion and dense clutter across the full 1–15 object range, providing a more reliable predictor for subsequent deployment on physical robots.
- In addition, we pointed out **in the conclusion** that the next step will be to transfer GR-AttNet to a real UR5 robotic arm in order to thoroughly evaluate its generalization capability and practical application value.

2. Additionally, the claimed 20ms inference for GR-CNN, which conflicts with their measured 200ms, warrants explanation.

**The author’s answer:**

- Thank you for your careful review of our work. To avoid any ambiguity, the sources of the two figures, the differences in testing methods, and the reasons for the tenfold discrepancy are explained below point by point: Kumra et al., in Section V-B of their original IROS-2020 paper (arXiv:1909.04810), reported that GR-CNN achieved an “average inference time of 19 ms” on an NVIDIA GTX-1080Ti. The authors subsequently clarified in the GitHub README (https://github.com/skumra/robotic-grasping, version dated 2021-04-27) that this timing only accounts for the **“GPU kernel time”**, that is, the portion of the model's forward pass (depth → tensors → grasp maps) enclosed by torch.cuda.synchronize(), and does not include: image reading, resizing, normalization, post-processing (angle inverse transformation, quality threshold filtering, NMS), the transfer of results from GPU back to CPU, as well as the overhead of the Python interpreter.
- Therefore, when reproducing Kumra et al.'s work locally, we adopted the same timing protocol as used for our method, which led to the observed discrepancy. We believe that the 200 ms discrepancy is not due to experimental error, but rather results from differing definitions between 'kernel time' and 'full pipeline time'. This point is detailed in the revised manuscript under “**GR-AttNet training results**”. **(See Lines 398 - 404 on Page 20 with highlight).**

3. Limited exploration of how the attention module generalizes to non-grasping robotics tasks?

**The author’s answer:**

- We sincerely appreciate your valuable suggestion regarding the lack of discussion on the generalization ability of attention modules to non-grasping robotic tasks. We fully agree that cross-task generalization is an important metric for evaluating the universal value of a module. However, as this paper focuses specifically on the problem of **“lightweight planar grasping in cluttered scenes”**, and due to limitations in experimental resources, we have not systematically conducted validation on non-grasping tasks.
- In the future, we plan to integrate the proposed attention mechanism module into networks such as the **R-FCN pose estimation backbone**. In addition, the independent attention units will be open-sourced for community verification.

4. Despite testing on Jacquard (synthetic) and Cornell (real) datasets, the model struggles with small objects or severe occlusions, as shown in Table 5, indicating limitations in the dataset. Possible to add real-world robot trials to validate simulation results?

**The author’s answer:**

- Thank you for this observation. We agree that the downward trend in success rates with increasing object numbers and occlusion **in Table 5** does reveal the gap between the current data and real-world scenarios. Due to experimental limitations, this paper has not yet conducted real-world experiments that correspond one-to-one with the simulations on an actual robotic platform, and therefore it is not possible to include a "real-robot" comparison table in the revised manuscript. We have truthfully stated in the **Conclusion (...In the future, we plan to deploy and validate on actual robotic platforms....).**
- Within the scope of the current work, the “real” validation we can provide includes:

- Cornell / Multi-Cornell real-world images (completely real acquisition);

- PyBullet physical simulation (collision, friction, mass, etc. parameters consistent with reality, but still a virtual environment).

- Once hardware support is available, we will perform real object grasping following the same 1→5→10→15 object layouts as in the simulations, and will open-source the data and code, reporting the complete results to the community at that time.

**Reviewer #2**:

Many thanks for your time and efforts in reviewing our manuscript. Your insightful comments have helped us significantly improve the quality of this paper. Our detailed reflections on your comments are provided below point by point.

1. First, some of the references are outdated. More recent works on transformer-based grasping and real-world deployment challenges from 2022–2024 should be discussed in depth.

**The author’s answer:**

- We appreciate the reviewer's valuable feedback on the timeliness of our references. In response, we have thoroughly revised the **"Related Work"** section to include a dedicated discussion of cutting-edge research from 2022-2024. Key additions include:
- An analysis of **transformer-based grasping methods**, such as the cross-modal fusion approach of Wang et al. [18] and the hybrid Mamba-Transformer architecture (HMT-Grasp) by Xiong et al. [20]. We discuss their impressive accuracy and the inherent trade-offs in computational complexity and real-time performance.
- This expansion not only updates the references but also provides a sharper contrast to highlight our contribution: a lightweight model that effectively balances accuracy, speed, and deployability.
- The added references are as follows: **(See References with highlight).**

[1] Jahanshahi H, Zhu ZH. Review of machine learning in robotic grasping control in space application. Acta Astronautica 2024;220:37-61.

[2] Jauhri S, Lunawat I, Chalvatzaki G. Learning Any-View 6DoF Robotic Grasping in Cluttered Scenes via Neural Surface Rendering. arXiv preprint arXiv:2306.07392. 2024 Feb 4.

[4] Kleeberger K, Bormann R, Kraus W, et al. A survey on learning-based robotic grasping[J]. Current Robotics Reports, 2020, 1(4): 239-249.

[15] Li M, Lu P, Zhu L, Zhu M, Zou L. Densely occluded grasping objects detection based on RGB-D fusion[J]. Control and Decision, 2023, 38(10): 2867-2874. DOI: 10.13195/j.kzyjc.2022.0259

[18]Wang Y, Li Y, Miao D, An C, Yuan X. Cross-modal interaction fusion grasping detection based on Transformer-CNN hybrid architecture[J]. Control and Decision, 2024, 39(11): 3607-3616. DOI: 10.13195/j.kzyjc.2023.1152

[20] Songsong Xiong, Hamidreza Kasaei. HMT-Grasp: A Hybrid Mamba-Transformer Approach for Robot Grasping in Cluttered Environments. arXiv preprint arXiv:2410.03522, 2024.

[22] Ainetter S, Fraundorfer F. End-to-end trainable deep neural network for robotic grasp detection and semantic segmentation from RGB. In: 2021 IEEE International Conference on Robotics and Automation (ICRA). Xi'an, May 30-Jun. 5, 2021.

[26] Paul SK, Chowdhury MT, Nicolescu M, Nicolescu M, Feil-Seifer D. Object Detection and Pose Estimation from RGB and Depth Data for Real-time, Adaptive Robotic Grasping. arXiv preprint arXiv:2101.07347. 2021 Jan 18.

2. Second, the paper suffers from redundancy and repeated explanations (e.g., the grasp pose definition is repeated almost verbatim).

**The author’s answer:**

We appreciate the reviewer's sharp eye in identifying issues of redundancy, such as the nearly verbatim repetition of the grasp pose definition. In response, we have undertaken a comprehensive revision to streamline the text:

I) **Grasp Pose Definition:** The duplicate explanation has been removed. We now present a unified and clear definition of the grasp pose representation (both in robot and image coordinates, along with the coordinate transformation) solely in the **"Grasp point definition"** subsection of the Methods. **(See Lines 141 - 169).**
II) **General Streamlining:** We have performed a line-by-line review to identify and eliminate other instances of repetitive explanations, particularly in the Introduction and Results sections, ensuring that each concept is introduced and discussed only once in its most logical location.

These changes significantly enhance the logical flow and conciseness of the manuscript.

3. Third, figures are referenced but not always explained in sufficient detail (e.g., the attention module in Fig. 2).

**The author’s answer:**

We appreciate the reviewer's feedback regarding the explanation of Fig. 2. We have addressed this by significantly enhancing the discussion of the attention module as follows:

I) **Detailed Architectural Explanation:** In the **"Attention block"** subsection of the **Methods**, we have now included a thorough, step-by-step analysis of the module depicted in Fig.2, explicitly linking each component in the diagram to its function and the corresponding equations. **(See Lines 242 - 273, Equations (4) to (7)).**
II) **Performance Validation in Results:** As the reviewer will find in the **"Results and Discussion"** section, we have already dedicated a substantial discussion to the module's performance. This includes the ablation studies in Table 2 that demonstrate the accuracy gains from different kernel combinations and a direct analysis of how the attention mechanism improves focus on target objects. **(See Lines 375 - 385).**

Together, these sections now provide a complete narrative: from the module's design to its empirical validation.

4. Fourth, the "Related Work" section could be improved by critically comparing the limitations of existing methods rather than simply listing them. The simulation results are promising, but they lack statistical robustness (e.g., there are no confidence intervals or discussions of variance across trials).

**The author’s answer:**

- We appreciate the reviewer's valuable feedback on enhancing the critical analysis in our related work and the statistical rigor of our results. We have addressed these concerns as follows:

I) **Critical Analysis in Related Work:** We have significantly revised the **"Related Work"** section. Instead of a sequential listing, it now organizes methods around key trade-offs (e.g., accuracy vs. speed, single-object vs. cluttered scenarios). We explicitly highlight the limitations of prior arts. For instance, we note that while Transformer-based methods achieve high accuracy, their computational complexity often hinders real-time deployment and that many high-performance models are primarily benchmarked on isolated objects, leaving a gap in clutter handling. This framing directly sets the stage for our contribution. **(See Lines 97 - 104, Lines 110 - 118, Lines 121 - 128 and Lines 129 - 138).**
II) **Statistical Robustness of Simulations**: To address the concern regarding statistical robustness, we have:

**· Calculated and reported the 95% confidence intervals (CIs) for all success rates in Table 5 using the Wilson score interval method, as recommended in statistical literature [41]. (See Lines 469 - 479 on Page 24).**

**·** Added a discussion in the **"Simulation results"** subsection that interprets these CIs, acknowledging the uncertainty in our estimates, especially as the number of objects increases.

**·** Explicitly stated in the revised manuscript that the results for the single-object scenario are derived from 50 trials, providing transparency about the sample size.

- These revisions substantially strengthen the scholarly contribution of the related work and the scientific validity of our experimental findings.

5. Lastly, there are some minor language and formatting issues that need correction, such as "Error! Reference source not found." and inconsistent terminology.

**The author’s answer:**

- We thank the reviewer for their meticulous review and valuable feedback. We have revised the manuscript to clarify these points and prevent any potential misunderstanding. All placeholder errors (e.g., **“Error! Reference source not found.”**) have been resolved, and all references, figures, and tables are now correctly linked and numbered.
- We have also performed a comprehensive polish of the manuscript's wording and phrasing to enhance the overall professionalism, clarity, and consistency of the academic expression.

**Reviewer #3**:

Many thanks for your time and efforts in reviewing our manuscript. Your insightful comments have helped us significantly improve the quality of this paper. Our detailed reflections on your comments are provided below point by point.

1. At line 31: There is an error in the reference not being found

**The author’s answer:**

Thank you for pointing out the issue of the missing reference at line 31. We have carefully checked and confirmed that the citation link is no longer valid, and **it has now been removed in the revised manuscript** to avoid causing confusion for readers.

2. At line 40: I believe there would be a reference for Kumra at the location of 'Kumra[] introduced the GR-CNN..."

**The author’s answer:**

- Thank you for reminding us to include the reference to Kumra at line 40. We have completed this in the revised manuscript as requested, now stated as: **Kumra et al. [9] later introduced RGB-D multi-modal fusion and residual skip connections in GR-CNN (Generative Residual Convolutional Neural Network)...**

3. At line 94: add a space between "closedloop" to be "closed loop"

**The author’s answer:**

- Thank you for highlighting the typographical error (“closedloop”) in line 94 of the original manuscript. In response to another reviewer's valuable comment regarding the timeliness of references in the Related Work section, we have substantially revised that paragraph. **The sentence containing the error was removed** as part of a comprehensive rewrite aimed at providing a more up-to-date discussion. Please review the related work again.

4. At lines 94-95: It states " enhancing the grasping ability for small objects, with a speed of 23 ms per frame and an accuracy increase to 97%. This is an increase from what or compared to what for small object grasping?

**The author’s answer:**

- Thank you for pointing out this ambiguity. We have revised the sentence to explicitly state that the accuracy increase to 97% refers to small-object grasping performance, compared to the baseline GR-CNN (92.3% on the small-object subset). This clarification is now included in the revised manuscript. **(See Lines 104 - 107 on Page 5)**.

5. At line 96: the same as the above what is the increasing detection accuracy for complex objects compared to. It states an increase to 86% but compared to what?

**The author’s answer:**

- Thank you for pointing out this ambiguity. We have revised the relevant sentence to clarify that **the detection accuracy increase to 86% refers to grasp detection performance on complex and occluded objects, compared to the baseline GR-CNN model without the attention mechanism.**
- Specifically, in multi-object cluttered scenes (e.g., heaps of tools like wrenches), the baseline model achieves 78% accuracy, while the integration of the CBAM-style attention module in Lu et.al’s method boosts this to 86%, representing an 8 percentage point improvement.
- This result highlights the effectiveness of the attention mechanism in suppressing background clutter and enhancing feature discrimination for complex-shaped or densely occluded objects. (See Lines 118 - 121 on Page 6).

6. At lines 105-108: Why are these lines the same as the lines 101-104 above equation 1? These lines 105-108 need removed.

**The author’s answer:**

- Thank you for pointing out the issue of duplicate content between lines 105–108 and lines 101–104. This was indeed an oversight on our part while organizing the manuscript. In the revised version, lines 105–108 **have been completely removed,** and the numbering of the subsequent paragraphs has been adjusted accordingly to ensure the text is concise and without redundancy **(See Lines 141 - 156 on Page 7)**.

7. At line 113 it states "height w and width h", I assume the w and h need switched so it states "height h and width w".

**The author’s answer:**

- Thank you for pointing out the typo in line 113, “height w and width h”. We have corrected it as per your suggestion to: **“...with height h and width w...”**We apologize for any confusion this may have caused. The revised manuscript has confirmed that all relevant statements are now consistent **(See Line 154 on Page 8 with highlight)**.

8. At line 158: It states this experiment only contains 2.8 million parameters. Please add what the standard model has for parameters. For example, this experiment only contains 2.8 million parameters while other models generally contain __ parameters.

**The author’s answer:**

- Thank you for pointing out the inaccuracy in the statement on line 158. We have now made the following revision: **The proposed network contains only 2.8 million parameters, compared to 35.2 million in the Oriented-Anchor FCN in [23] and 46.7 million in the ConvNet-Grasp in [24], making it significantly more lightweight. (See Line 218-220 on Page 11 with highlight).**

9. At lines 175 and 176: I just want a change here for clarity. I want it changed to state that "The input feature map V is first convolved with two kernels of size 3x3 and 7x7." I believe there are only two and the wording of kernels of sizes 3x3 and 7x7 makes it seem that there are more than 2.

**The author’s answer:**

- Thank you for the clarification. We have updated lines 175–176 to: **“The input feature map V is first convolved with two kernels of size 3×3 and 7×7.”** This wording now clearly indicates that only two kernels are used **(See Line 244-245 on Page 12 with highlight)**.

10. At line 176: Did you mean to state that the convolution kernels will be discussed in detail in Conclusion? The conclusion section did not appear to discuss this in detail and the Results sections has this discussion.

**The author’s answer:**

- We thank the reviewer for this helpful observation. The mention of the "Conclusion" section was an oversight from an earlier draft. In the revised manuscript, we have reorganized the paper structure by establishing a dedicated **"Results and Discussion" section**. As the reviewer rightly suggested, the detailed discussion regarding the selection of convolutional kernels is now comprehensively presented in the new subsection titled **"a) Attention mechanism results"(See Line 356-385 on Page 19 with highlight)** within this section.

11. Add a Training section to the Methods and move lines 195 to 224 to this new training section at the end of the Methods

**The author’s answer:**

- We thank the reviewer for this constructive suggestion. Accordingly, **we have added a new "Training" subsection** to the **"Materials and Methods" section**. The content previously located in lines 195-224 has been moved to this new subsection, as the reviewer recommended. **(See Line 274 on Page 14 with highlight)** This reorganization improves the logical flow and clarity of the Methods.

12. Change the Section "Training and Results" to "Results and Discussion" I see that you did not have a discussion section and it appears that you are having it with the results.

**The author’s answer:**

- We thank the reviewer for this helpful suggestion. We agree that integrating the discussion with the results improves the flow of the manuscript. Accordingly, we have changed the section title from **"Training and Results"** to **"Results and Discussion"** throughout the paper. **(See Line 354 on Page 18 with highlight).**

13. At line 240: states "has only 2.8M parameters, making it more deployable." More deployable compared to what? Add what it is more deployable than.

**The author’s answer:**

- We thank the reviewer for pointing out this lack of clarity. The statement was intended to highlight the advantage of our model against larger, more complex counterparts. We have revised the sentence to explicitly state the comparison. It now clearly reads that our model is "more deployable than similar grasping prediction techniques that utilize complex architectures and involve millions of parameters," thereby emphasizing its lower computational cost and suitability for edge deployment.
- Furthermore, to further strengthen the argument for the deployability of our model with only 2.8M parameters, we have provided a comprehensive explanation in the revised manuscript. **(See Lines 390-394 on Page 20 with highlight).**

14. At lines 242: It states that the baseline method... Is the baseline method GR-CNN's? Be more specific here for what the baseline method is.

**The author’s answer:**

- We thank the reviewer for this precise feedback. We agree that the term "baseline method" was ambiguous. We have revised the sentence at line 242 (and in the corresponding context) to explicitly state that **the baseline method is "GR-CNN"** to avoid any confusion.**(See Line 396 on Page 20 with highlight).**

15. At lines 242 and 243: It states that the baseline method has a theoretical inference speed of 20 ms but you found that it actually ran at 200 ms. Add why there was a time difference. Why did it take 200 ms instead of 20 ms. Was it a hardware issue or something?

**The author’s answer:**

- Thank you for your careful review of our work. To avoid any ambiguity, the sources of the two figures, the differences in testing methods, and the reasons for the tenfold discrepancy are explained below point by point: Kumra et al., in Section V-B of their original IROS - 2020 paper (arXiv:1909.04810), reported that GR-CNN achieved an “average inference time of 19 ms” on an NVIDIA GTX-1080Ti. The authors subsequently clarified in the GitHub README (https://github.com/skumra/robotic-grasping, version dated 2021-04-27) that this timing only accounts for the **“GPU kernel time”**, that is, the portion of the model's forward pass (depth → tensors → grasp maps) enclosed by torch.cuda.synchronize(), and does not include: image reading, resizing, normalization, post-processing (angle inverse transformation, quality threshold filtering, NMS), the transfer of results from GPU back to CPU, as well as the overhead of the Python interpreter.
- Therefore, when reproducing Kumra et al.'s work locally, we adopted the same timing protocol as used for our method, which led to the observed discrepancy. We believe that the 200 ms discrepancy is not due to experimental error, but rather results from differing definitions between 'kernel time' and 'full pipeline time'. This point is detailed in the revised manuscript under **“GR-AttNet training results”**. **(See Lines 398 - 404 on Page 20 with highlight).**

16. At line 244: Add something to discuss what the 148 ms and 163 ms means in comparision to the benchmark that takes 200 ms. It appears that this would be important for practically applying it.

**The author’s answer:**

- We thank the reviewer for this valuable suggestion. We agree that discussing the practical implication of the inference speed is crucial. Accordingly, we have added a discussion in the revised manuscript (in the same paragraph) that explicitly compares our speeds (148 ms and 163 ms) against the 200 ms benchmark. The added text explains that our method's faster processing is a significant advantage for real-time applications, as it provides a larger margin to meet the strict latency requirements of dynamic tasks like conveyor belt picking or human-robot interaction.**(See Lines 405 - 409 on Page 20 with highlight).**

17. At line 251: Add a space between "highperformance" to be "high performance".

**The author’s answer:**

- We thank the reviewer for catching this typographical error. The correction has been made, and **"highperformance"** has been changed to **"high performance"** in the revised manuscript. **(See Line 418 on Page 21 with highlight).**

18. At line 251: Add something regarding discussion if possible for how the GR-AttNet improves over other high performance models

**The author’s answer:**

- We thank the reviewer for this valuable suggestion to strengthen the discussion. We have added the following analysis in the revised manuscript:
- "**While GR-AttNet achieves comparable accuracy to other high-performance models, its key advantage lies in the balanced trade-off between accuracy, parameter efficiency, and inference speed.** Compared to large-scale architectures like Oriented-Anchor FCN [23] (35.2M parameters) and Transformer-based methods that achieve higher accuracy but at significantly higher computational cost, GR-AttNet maintains competitive performance with only 2.8M parameters. This parameter efficiency, combined with the attention mechanism's ability to focus on relevant spatial features, enables more robust performance in cluttered scenarios where other models typically struggle. The linear integration of the attention module allows this improvement without substantially increasing architectural complexity, making GR-AttNet particularly suitable for real-world applications where computational resources are constrained."**(See Lines 419–424 on Page 21 with highlight).**

19. Regarding FIG. 4 - Add the Q like in FIG. 3 to show the final grasping quality map.

**The author’s answer:**

- We thank the reviewer for this suggestion. We have updated Fig. 4 as recommended by **adding the final grasping quality map** (Q) to the visual results, making it consistent with the presentation in Fig. 3. The revised figure now provides a more complete visualization of the network's output. **(See Fig.7 and Fig.8 on Page 23).**

20. After the new training section you added in the methods add a new section for the simulation environment setup and move lines 271-299 moving FIGS. 5 and 6 to the methods section. Most likely requiring renumbering some FIGS. This section appears to be methods of what was done.

**The author’s answer:**

- We thank the reviewer for this excellent suggestion regarding the organization of the manuscript. We agree that the simulation environment setup describes the experimental methodology. Accordingly, we have created a new subsection titled **"Simulation Environment Setup"** within the **"Materials and Methods"** chapter, placed after the **"Training"** subsection. The content from the original lines 271-299, along with Figures 5 and 6 **(now renumbered as Figures 3 and 4 within the Methods section)**, has been moved to this new subsection. All subsequent figures and in-text citations have been updated accordingly. **(See Fig.3, Fig.4 and Lines 347 - 351 on Page 17 ).**

21. Since the text above Table 5 was moved to the methods. Add a small amount of text to introduce the Table 5 if desired. Could move some of the results discussed below the Table to above.

**The author’s answer:**

- We are grateful to the reviewer for this valuable comment. To better frame the results, we have introduced **a succinct description of the simulation experiment** above Table 5. This addition, which briefly outlines the experimental protocol (e.g., number of trials and failure conditions), offers readers the necessary context to interpret the subsequent data, significantly improving the flow and completeness of the section **(See Lines 453–457 on Page 23).**

22. Add a paragraph or two to be more of a discussion of Table 5 after line 318. Something regarding how the grasping result shown in Table 5 relates to other high performance models and any next steps based on the result.

**The author’s answer:**

- We sincerely thank the reviewer for the valuable suggestion to strengthen the discussion of **Table 5**. In response, we have added a new discussion paragraph that builds directly on the provided context. This addition makes two important contributions: first, it explicitly contrasts the performance trends observed in our multi-object simulations with the high success rates reported by other models on single-object benchmarks **(e.g., Lou [25] and GG-CNN [8])**, underscoring that our study addresses a critical gap in evaluating grasping under realistic, cluttered conditions. Second, it clearly links the failure modes identified in the table—such as slippage and occlusion errors—to specific future research directions, emphasizing our planned work on enhancing physical interaction modeling and occlusion reasoning.
- In addition, to further reinforce the validity of our findings, we have included extensive ablation studies in the revised manuscript and applied Wilson score intervals to quantify the statistical confidence of all reported success rates. These enhancements provide a more rigorous and reliable evaluation of our method’s performance. **(See Lines 480–484 on Page 24 with highlight).**

23. At lines 396-398 - I believe this is some template text. Adding a link or something to the data underlying the results needs added here. I believe this is a requirement to make the underlying data accessible.

**The author’s answer:**

- We thank the reviewer for pointing this out. The text in lines 396-398 was indeed a placeholder for the Data Availability Statement. We have now replaced it with a complete statement that includes the direct URLs from which the Cornell and Jacquard datasets can be accessed, as well as the DOI link to our own code repository on GitHub, thus fully complying with the data accessibility requirements **(See Lines 520–524 on Page 26).**

Our hope is that these reflections will meet your approval. Thanking you for your valuable time and constructive comments.

Sincerely,

*The Authors*
